# CDC20 maintains tumor initiating cells

**DOI:** 10.18632/oncotarget.3676

**Published:** 2015-04-28

**Authors:** Qi Xie, Qiulian Wu, Stephen C. Mack, Kailin Yang, Leo Kim, Christopher G. Hubert, William A. Flavahan, Chengwei Chu, Shideng Bao, Jeremy N. Rich

**Affiliations:** ^1^ Department of Stem Cell Biology and Regenerative Medicine, Lerner Research Institute, Cleveland Clinic, Cleveland, OH 44195, USA; ^2^ Department of Molecular Medicine, Cleveland Clinic Lerner College of Medicine of Case Western Reserve University, Cleveland, OH 44195, USA; ^3^ Department of Pathology and Center for Cancer Research, Massachusetts General Hospital and Harvard Medical School, Boston, MA 02114, USA; ^4^ Broad Institute of Harvard and Massachusetts Institute of Technology (MIT), Cambridge, MA 02142, USA; ^5^ Howard Hughes Medical Institute, Chevy Chase, MD 20815, USA; ^6^ Division of Neurosurgery, Department of Surgery, Kaohsiung Medical University Hospital, Kaohsiung 80756, Taiwan

**Keywords:** cancer stem cell, glioblastoma, glioma, tumor initiating cell, CDC20

## Abstract

Glioblastoma is the most prevalent and lethal primary intrinsic brain tumor. Glioblastoma displays hierarchical arrangement with a population of self-renewing and tumorigenic glioma tumor initiating cells (TICs), or cancer stem cells. While non-neoplastic neural stem cells are generally quiescent, glioblastoma TICs are often proliferative with mitotic control offering a potential point of fragility. Here, we interrogate the role of cell-division cycle protein 20 (CDC20), an essential activator of anaphase-promoting complex (APC) E3 ubiquitination ligase, in the maintenance of TICs. By chromatin analysis and immunoblotting, CDC20 was preferentially expressed in TICs relative to matched non-TICs. Targeting CDC20 expression by RNA interference attenuated TIC proliferation, self-renewal and *in vivo* tumor growth. CDC20 disruption mediated its effects through induction of apoptosis and inhibition of cell cycle progression. CDC20 maintains TICs through degradation of p21^CIP1/WAF1^, a critical negative regulator of TICs. Inhibiting CDC20 stabilized p21^CIP1/WAF1^, resulting in repression of several genes critical to tumor growth and survival, including CDC25C, c-Myc and Survivin. Transcriptional control of CDC20 is mediated by FOXM1, a central transcription factor in TICs. These results suggest CDC20 is a critical regulator of TIC proliferation and survival, linking two key TIC nodes – FOXM1 and p21^CIP1/WAF1^ — elucidating a potential point for therapeutic intervention.

## INTRODUCTION

Glioblastoma ranks among the most lethal human cancers with current therapies offering only palliation [1]. Like other solid tumors, glioblastomas phenocopy aberrant organ systems with heterogeneity within the neoplastic compartment derived from genetic and epigenetic causes, leading to cellular hierarchies with self-renewing TICs at the apex [2, 3]. TICs have generated a substantial interest due to their resistance to conventional therapies, evasion of anti-tumor immune responses, promotion of tumor angiogenesis and invasion [4, 5, 6, 7, 8, 9, 10]. Although the molecular regulation of TICs has been largely informed by the application of core regulatory signaling in normal embryonic and tissue-specific stem cells, we and others have identified key, targetable nodes in TICs, including NOS2, BMX, GLUT3, EphA2, transforming growth factor-β, and FOXM1 [11, 12, 13, 14, 15, 16, 17], indicating that there are distinct signaling pathways controlling TICs. Recent studies have also demonstrated that TICs have specific regulation of mitotic control [18].

Ubiquitination-mediated protein degradation critically regulates TICs [19, 20], so we investigated the role of the function of a key E3 ligase, CDC20, in the maintenance of TICs. CDC20 is a WD40 repeat domain-containing protein that mediates the activation of the anaphase-promoting complex (APC) E3 ubiquitination ligase. CDC20-APC recognizes the D-box or KEN box of substrates to promote proteosomal degradation [21]. Multiple cancer types display increased CDC20 expression [22, 23, 24, 25], but the role of CDC20 in glioblastoma generally and TICs, in particular, is unclear. Here, we demonstrate a novel function of CDC20 mediating TIC proliferation, self-renewal and tumor growth by connecting two key TIC nodes, FOXM1 and p21^CIP1/WAF1^. Collectively, these findings demonstrate a new signaling pathway in TIC maintenance and provide a novel target for therapeutic development to improve GBM treatment.

## RESULTS

### TICs preferentially express CDC20

**Figure 1:**
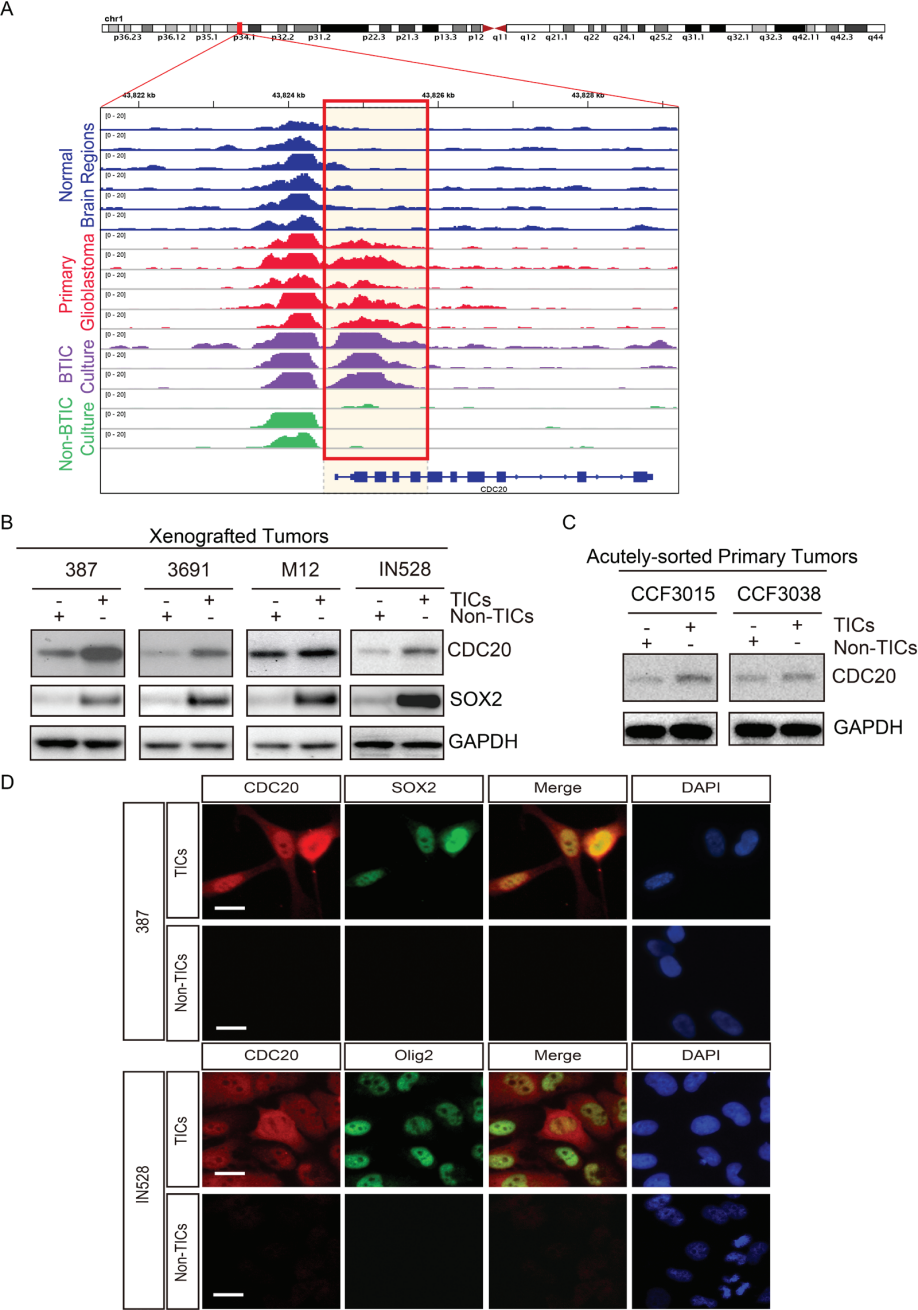
CDC20 is highly expressed in tumor initiating cells (TICs) **A.** H3K27ac ChIP-seq enrichment plot centered at the CDC20 gene locus. Enrichment is shown for various normal brain regions (blue, Roadmap Epigenomics Project, Ref.26), a series of five primary glioblastomas (red), glioblastoma TICs (purple, *n* = 3; Ref. 27), and differentiated glioblastoma cells (green, *n* = 3; Ref. 27). The orange box highlights a transcriptionally active region found exclusively in primary glioblastomas and TICs. **B.** Immunoblot analysis of CDC20 protein levels in glioblastoma TICs and non-TICs isolated from patient-derived xenografts (387, 3691, M12 and IN528). **C.** Immunoblot analysis of indicated proteins in TICs and non-TICs derived from two primary human GBM specimens without culture (CCF3015, CCF3038). **D.** Immunofluorescent staining of CDC20 with several TIC markers including SOX2 and OLIG2.

CDC20 is an important regulator of the cell cycle so we performed an *in silico* analysis of CDC20 expression in glioma patients. CDC20 was highly expressed in glioblastomas, relative to normal brain and lower grade glioma (Supplemental Figure 1A). Higher CDC20 expression correlated with shorter survival of glioma patients, befitting its association with tumor grade (Supplemental Figure 1B). As TICs are highly enriched in high-grade gliomas, CDC20 may play an important role in the maintenance of TICs. The differentiation state of a cell is reflected in its chromatin regulation so we investigated CDC20 enhancer regulation through the interrogation of the acetylation status of histone H3 (H3K27ac), a mark associated with active transcription. We performed H3K27ac chromatin immunoprecipitation combined with high-throughput sequencing (ChIP-seq) of a series of glioblastoma surgical specimens immediately after resection in the absence of culture then compared CDC20 regulation with similar analyses performed on regions of normal brain (Roadmap Epigenomics Project) [26] and three glioblastoma lines separated into TICs and differentiated progeny and deposited *in silico* [27], revealing that patient glioblastomas and TICs have active CDC20 enhancers, whereas normal brain and non-TICs do not (Figure 1A). To investigate the function of CDC20 in TIC biology, we examined the expression of CDC20 in functionally validated TICs and matched non-TICs from patient-derived xenografts by immunoblotting (Figure 1B). While segregation of TICs from non-TICs is an area of substantial controversy, we selected validated models and methods to separate self-renewal and tumor initiation [11, 12, 13]. In each comparison of TICs and non-TICs we tested, TICs displayed strikingly elevated CDC20 protein levels relative to matched non-TICs. To rule out any effect caused by culture conditions, we confirmed these results using TICs and non-TICs directly isolated from primary GBM patient specimens without culture (Figure 1C). To broaden the evidence to other TIC markers, we performed immunofluorescent staining and found that CDC20 was co-expressed with TIC markers SOX2 and OLIG2, confirming marker independent TIC expression of CDC20 (Figure 1D).

### CDC20 is necessary for TIC maintenance

**Figure 2:**
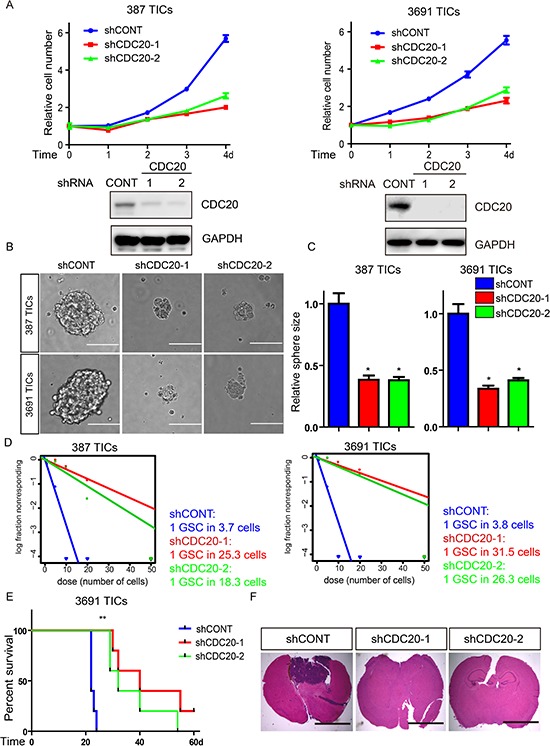
Targeting CDC20 by RNA interference decreases TICs growth, self-renewal, and tumor formation **A.** Top: Effects of CDC20 knockdown with two independent shRNA constructs on cell proliferation in two TIC lines. Bottom: Immunoblots of CDC20 following knockdown via shRNAs in two TIC lines. **B.** Representative images of tumorspheres derived from two TIC lines expressing control shRNA (shCONT), shCDC20-1, or shCDC20-2 are shown. Scale bars indicate 100 μm. **C.** Quantification shows reduced tumorsphere size knockdown DRP1 (*p < 0.05; *n* = 3). **D.** *In vitro* extreme limiting dilution assays (ELDA) demonstrate that knockdown of CDC20 in two TIC lines decreases the frequency of tumorsphere formation. **E.** Kaplan-Meier survival curves of immunocompromised mice bearing intracranial 3691 TICs expressing shCONT, shCDC20-1, or shCDC20-2 (**p < 0.01; *n* = 5). **F.** Representative images of cross sections (hematoxylin and eosin stained) of mouse brains harvested on day 22 after transplantation of 3691 TICs expressing shCONT, shCDC20-1, or shCDC20-2.

We next interrogated the requirement for CDC20 function in TIC maintenance. We developed two independent, non-overlapping small hairpin RNA (shRNA) lentiviral constructs to knockdown CDC20 (designated hereafter as shCDC20-1 and shCDC20-2) and compared their effects to a control shRNA insert (shCONT) that does not target any known genes from any species, making it useful as a negative control against nonspecific effects. Knockdown efficiency was confirmed by immunoblot (Figure 2A, bottom). We then examined the phenotypic consequences of shRNA-mediated reduction of CDC20 expression. Silencing CDC20 significantly decreased the growth of TICs (Figure 2A, top), supporting the requirement of CDC20 for TIC growth. To test whether targeting CDC20 influences tumorsphere formation (a surrogate marker of self-renewal), we performed *in vitro* limiting dilution assays with TICs expressing non-targeting control shRNA or CDC20-directed shRNAs. CDC20 knockdown resulted in a more than fivefold decrease in the frequency of sphere formation and a greater than twofold decrease in the sphere size (Figure 2B–2D). The most important property of TICs is their potent ability to form tumors *in vivo*. To address the requirement for CDC20 in maintaining the tumorigenic potential of TICs, we examined the effects of CDC20-directed interventions *in vivo*. TICs transduced with lentivirus encoding either of two non-overlapping CDC20-targeting shRNAs or control shRNA were transplanted into the brains of immunocompromised mice. Animals bearing TICs expressing shCDC20 showed significantly reduced tumor formation and increased survival relative to control tumors bearing TICs expressing control shRNA (Figure 2E, 2F), supporting CDC20 as necessary to maintain the tumorigenic potential of TICs. Taken together, our findings demonstrate that CDC20 downregulation attenuates TIC phenotypes including proliferation, self-renewal, and tumor formation.

### CDC20 inhibition induces cell cycle arrest and apoptosis in TICs

**Figure 3:**
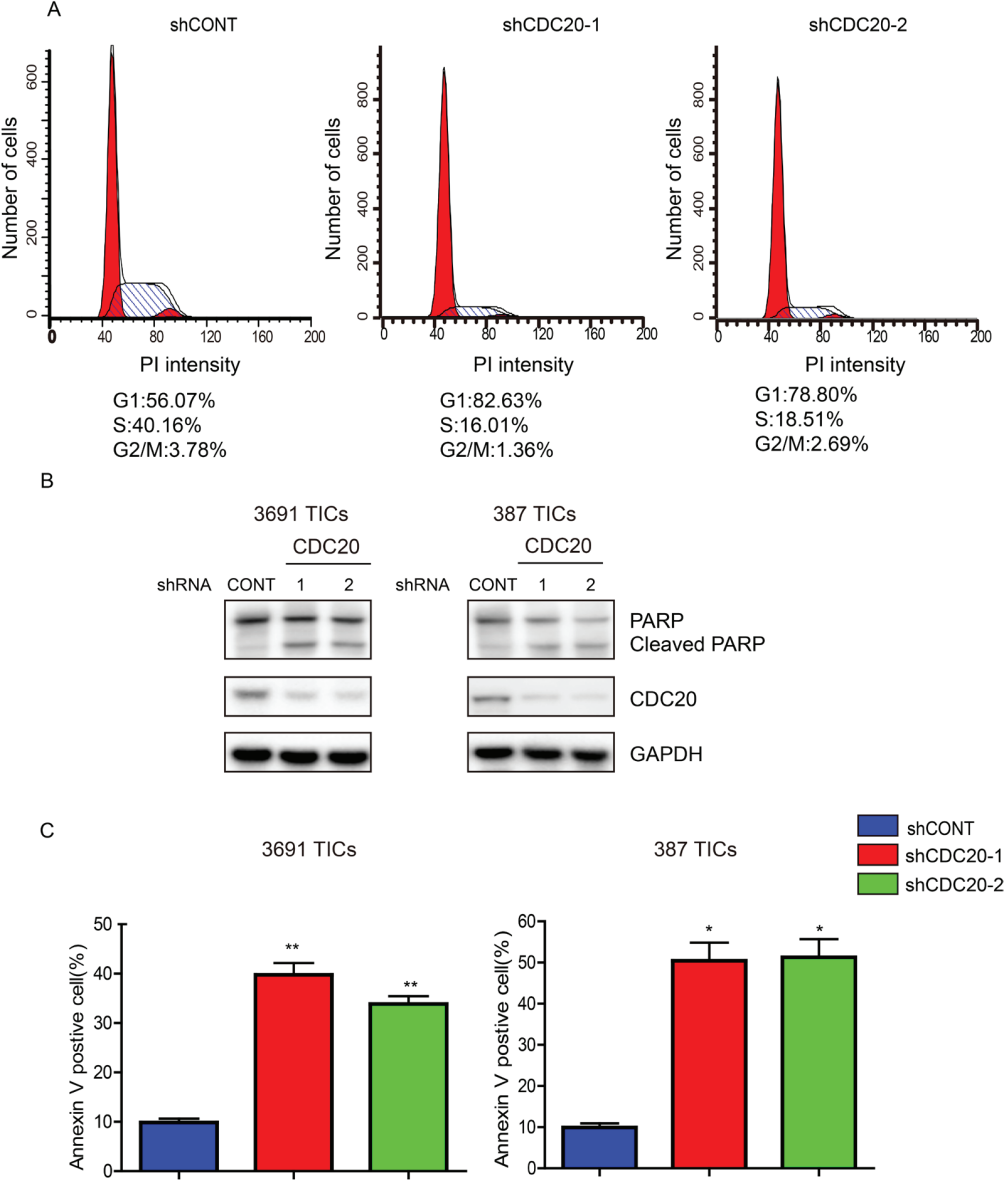
CDC20 Depletion Induces cell cycle arrest and apoptosis of TICs **A.** Cell cycle analysis of 3691 TICs expressing control shRNA (shCONT), shCDC20-1, or shCDC20-2. **B.** Lysates of 3691 and 387 TICs expressing shCONT or shCDC20 were immunoblotted with the indicated antibodies. shRNA-mediated knockdown of CDC20 increased cleaved PARP. **C.** Apoptosis measured by AnnexinV staining in 3691 and 387 TICs expressing shCONT or shCDC20. Data are presented as mean ± SEM (*p < 0.05; **p < 0.01).

CDC20 is a central regulator of the cell cycle in numerous cancers [24, 28]. Based on this function, we interrogated the cell cycle following CDC20 knockdown. Concordant with the decrease in tumor growth, we found after TIC transduction with shCDC20, CDC20 knockdown caused loss of TICs in the S, M and G_2_ cell cycle phases and accumulation in the G_1_ phase (Figure 3A). In addition, we observed a significant increase in apoptotic cell death after silencing CDC20 expression in TICs, as measured by both cleaved PARP and Annexin V staining (Figure 3B, 3C). Collectively, these results demonstrate that CDC20 serves not only in the cell cycle progression of TICs, but also promotes the survival of TICs.

### CDC20 regulates p21^WAF1/CIP1^ in TICs

**Figure 4:**
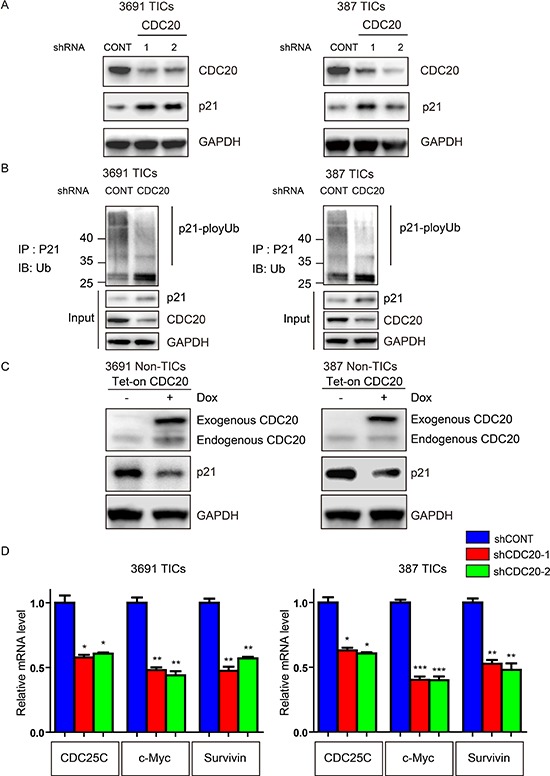
CDC20 negatively regulates p21^WAF1/CIP1^ in TICs **A.** Lysates of 3691 and 387 TICs expressing control shRNA (shCONT), shCDC20-1, or shCDC20-2 were immunoblotted with the indicated antibodies. shRNA-mediated knockdown of CDC20 increased p21^WAF1/CIP1^ protein levels. **B.** Lysates of 3691 and 387 TICs expressing shCONT or shCDC20 were immunoprecipitated with a p21^WAF1/CIP1^ antibody and then immunoblotted with the indicated antibodies. Knockdown of CDC20 decreased p21^WAF1/CIP1^ ubiquitylation. **C.** 3691 and 387 NSTCs were transduced with a Tet-on CDC20 lentiviral vector with puromycin selection (1 μg/ml) for 72 hours. Surviving cells were treated with 500 ng/ml doxycycline or vehicle control (DMSO) for 48 hours. Cells were lysed and immunoblotted with the indicated antibodies. **D.** 3691 and 387 TICs were infected with CDC20 shRNAs or control vector for 2 days. Total RNA was isolated and cDNA was synthesized by reverse transcription. The mRNA levels of indicated genes were detected by real-time qPCR (*p < 0.05; **p < 0.01; ***, *p* < 0.001; *n* = 3).

To determine potential downstream mediators of CDC20, we considered key TIC regulators that have been also linked to CDC20. As CDC20 has been shown to control p21^WAF1/CIP1^ protein levels through ubiquitin-mediated degradation [29] and p21^WAF1/CIP1^ is an essential negative regulator in TICs [30, 31, 32, 33], we examined CDC20 regulation of p21^WAF1/CIP1^ in TICs. Disruption of CDC20 significantly reduced p21^WAF1/CIP1^ protein levels, but not mRNA levels in TICs (Figure 4A and Supplemental Figure S2). Consistent with CDC20-APC ubiquinylation function, targeting CDC20 reduced p21^WAF1/CIP1^ poly-ubiquitination (Figure 4B). In a gain-of-function approach, CDC20 overexpression in non-TICs decreased p21^WAF1/CIP1^ expression (Figure 4C). p21^WAF1/CIP1^ functions not only as a CDK inhibitor, but also as a transcriptional co-repressor of several important genes controlling cell fate. Indeed, CDC20 knockdown led to decreased expression of p21^WAF1/CIP1^ downstream target genes (Figure 4D). These genes not only included cell cycle regulators (CDC25C), but also core stem cell regulators (c-Myc) and anti-apoptotic mediators (Survivin), which is consistent with our findings in CDC20 function. These data support p21^WAF1/CIP1^ as an essential downstream target of CDC20 in regulation of proliferation, self-renewal and cell survival of TICs.

### FOXM1 regulates CDC20 in TICs

**Figure 5:**
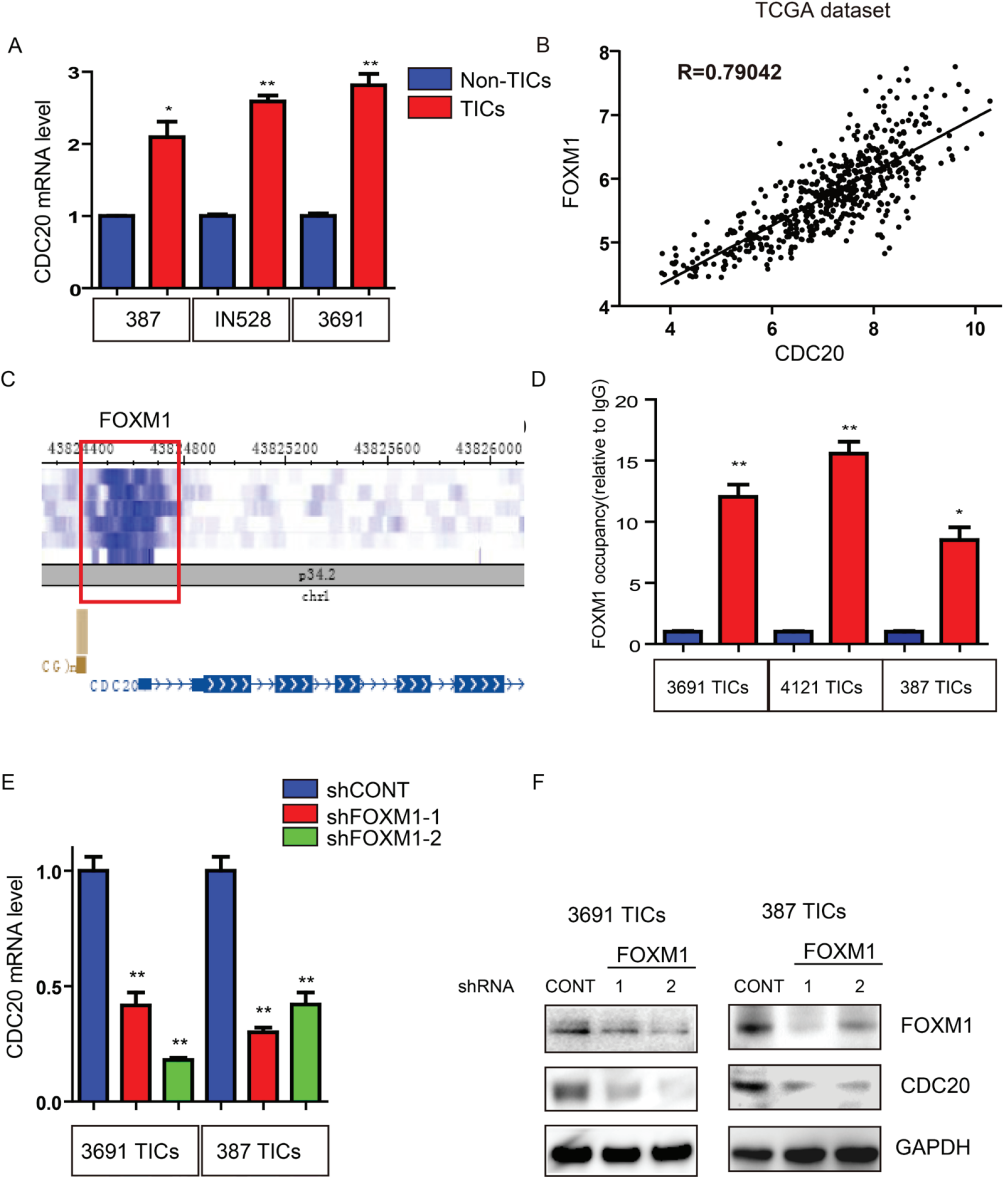
Transcription factor FOXM1 upregulates CDC20 expression in TICs **A.** CDC20 mRNA levels in TICs and non-TICs were detected by real-time qPCR. Data are displayed as mean ± SEM (*p < 0.05; **p < 0.01; *n* = 3). **B.** The TCGA GBM dataset was downloaded and correlations analyzed by R. FOXM1 and CDC20 levels were highly correlated. **C.** FOXM1 is enriched in the promoter region of CDC20 (from WashU EpiGenome Brower). **D.** Cross-linked chromatin was prepared from three TIC lines then immunoprecipitated with an anti-FOXM1 antibody or IgG control followed by real-time PCR using primers specific to CDC20 promoter (*p < 0.05; **p < 0.01; *n* = 3). **E.** CDC20 mRNA levels in 3691 and 387 TICs transduced with control shRNA (shCONT) or FOXM1 shRNAs were detected by real-time qPCR. Data are displayed as mean ± SEM (**p < 0.01; *n* = 3). **F.** Lysates of 3691 and 387 TICs expressing - shCONT, shFOXM1–1, or shFOXM1–2 were immunoblotted with the indicated antibodies. shRNA-mediated knockdown of FOXM1 decreased CDC20 levels.

To determine the upstream mechanism driving CDC20 expression in TICs, we interrogated the level at which TICs showed relative expression differences. Consistent with our findings of preferential enhancer regulation of CDC20 in TICs (Figure 1A), real-time qPCR showed that CDC20 mRNA levels are upregulated in TICs, indicating a potential role of transcriptional activators in regulating CDC20 expression (Figure 5A). We therefore performed an *in silico* analysis of the TCGA GBM expression dataset to discover transcription factors whose expression was strongly correlated with CDC20. The top hit was FOXM1, a central regulator of GBM and TICs [14, 34, 35, 36], with an R-value of 0.79 (Figure 5B). As independent confirmation, we found that FOXM1 was enriched in a promoter region of CDC20 probing the publicly available CHIP-seq database, WashU EpiGenome Brower (Figure 5C). To translate these findings into direct experimental analysis using our TICs, we performed ChIP for FOXM1 in TICs and confirmed binding of FOXM1 to the CDC20 promoter in TICs by CHIP-PCR (Figure 5D). Moreover, silencing FOXM1 significantly reduced CDC20 mRNA and protein expression in TICs (Figure 5E, 5F). Collectively, our data demonstrate that FOXM1 directly activates CDC20 to facilitate transcription in TICs.

## DISCUSSION

Organs with clearly defined cellular hierarchies in development and homeostasis – blood, brain, breast, skin, and colon – give rise to tumors with defined cellular hierarchies, suggesting that tumors recapitulate organ systems mimicking their origin [37]. The ability to prospectively distinguish TICs, which reside at apex of tumor hierarchies, from their differentiated progeny remains challenging; however, stem cell biology faces similar difficulty with normal stem cell identification, especially in human tissues. Cell surface markers mediate interactions between a cell and its microenvironment. The dissociation of cells from their surroundings induces a rapid degradation of informational content of markers, requiring rapid utilization of these markers. Most TIC markers have been appropriated from normal stem cells, but the linkage between TICs and normal stem cells remains controversial. Conceptually, the cancer stem cell hypothesis does not claim a stem cell as the cell-of-origin for cancers, but the precise relationship between TICs and normal stem cells remains under active investigation.

Normal neural stem cells have generally been considered quiescent [38, 39, 40], while brain cancer cells display dysregulated cellular proliferation, leading some investigators to question the validity of the cancer stem cell hypothesis. However, several lines of evidence suggest that this division between normal and neoplastic stem cell regulation may not be as distinct as initially conceived. Embryonic stem cells rapidly progress through the cell cycle without checkpoints [41], and many components of the core embryonic stem cell machinery – SOX2, OCT4, NANOG, and c-Myc – are expressed and active in cancer stem cells [32, 42, 43, 44]. Leukemia initiating cells display relative quiescence, phenocopying hematopoietic stem cells [45]. The proliferative potential of solid tumor stem cells is less established, but recent studies of a number of organs, notably the colon and skin, have shown that these organs often contain at least two distinct stem cell compartments, one proliferative and one quiescent [46, 47]. As been one of the most reliable solid tumors for studies of the cellular hierarchy, glioblastoma offers an excellent platform to investigate TIC proliferation. Genetically engineered mouse models demonstrate that glioblastomas may originate not only from quiescent neural stem cells, but also their proliferative progeny, oligodendroglial progenitors, suggesting that TICs may share a proliferative potential with these progenitors [48, 49, 50, 51]. Single cell analyses of glioblastomas directly from patients have demonstrated dramatic variation in tumor cell genetics, gene expression, growth patterns, and sensitivity to treatments [52]. In standard stem cell conditions with high concentrations of growth factors, glioma TICs are highly proliferative, but TIC cultures also contain label-retaining cells that can be highly tumorigenic and resistant to conventional therapies [53]. Direct analysis of glioblastoma patient specimens for TIC and proliferation markers demonstrates variability in the prevalence of proliferating TICs with an inverse relationship to prognosis [54]. Although TICs are often resistant to chemotherapies that preferentially target proliferative cells, specifically targeting mitotic control may offer novel treatment strategies with a significant therapeutic index. For example, a RNA interference screen of glioma TICs demonstrated critical dependence of TICs on mitotic control [55].

**Figure 6:**
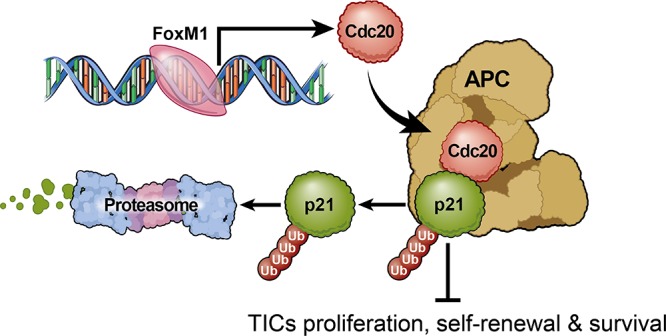
Proposed model of CDC20 function in tumor initiating cells

In the current studies, we interrogated mitotic control of TICs through a combination of direct experimental and *in silico* studies to uncover a novel signaling pathway, FOXM1-CDC20-p21^WAF1/CIP1^, which maintains TIC self-renewal and tumorigenic potential (modeled in Figure 6). As CDC20 is oncogenic in a number of human cancers, including breast cancer, cervical cancer, and gastric cancers [25, 56, 57], CDC20 may regulate TICs in other cancers. Previous reports of CDC20 expression in glioma demonstrated a positive correlation tumor grade [58, 59], similar to our *in silico* findings. However, the biological significance of CDC20 in GBMs, and TICs, in particular, has been poorly understood. Our findings strongly indicate that CDC20 is a pro-oncogenic gene in GBM growth, as CDC20 plays an essential role in the regulation of TIC proliferation, self-renewal and survival.

Most studies of CDC20 had focused its function and its downstream ubiquitin targets, but the upstream regulators of CDC20 have been largely uninvestigated. Kidokoro and co-workers demonstrated that p53 binds to the promoter of CDC20 to inhibit its expression, suggesting that p53 as a negative regulator of Cdc20 [57]. Here, we identified FOXM1 as a transcriptional activator of CDC20. FOXM1 directly binds to the promoter of CDC20 to induce its transcription while shRNA-mediated silencing of FOXM1 decrease CDC20 expression in TICs. FOXM1 has been well understood as a key regulator of TICs through a number of targets [14, 35, 36], including recent reports from Nakano and co-workers that maternal embryonic leucine zipper kinase (MELK) phosphorylates FOXM1 to regulate radioresistance [35, 36]. While Huang and co-workers found that heat shock factor 1 (HSF1) regulates FOXM1 in glioma with changes in cell cycle proteins, including CDC20 [34], our results provides the first direct evidence that FOXM1 maintains TICs via regulation the expression of CDC20.

In our studies, we found that p21^WAF1/CIP1^ was a major downstream target of CDC20 in controlling TIC proliferation, self-renewal and survival. It is probable that other CDC20 targets are also involved in mediating the effects of CDC20 in TICs, serving as a possible focus for future studies. p21^WAF1/CIP1^ has been defined as an essential negative regulator in TICs [14, 15, 16, 17]. In neural stem cells, p21^WAF1/CIP1^ promotes self-renewal through a number of different mechanisms [19, 20]. The differential effects of p21^WAF1/CIP1^ between TICs and neural stem cells indicate that CDC20 might be a potential specific target for TICs with acceptable toxicity against normal brain.

Recently, Wan and colleagues demonstrated that the CDC20-APC complex represses cell apoptosis through targeting Bim ubiquitination and degradation [60]. In our study, we also found cell apoptosis induced by CDC20 knockdown, but without significant induction of BIM (data not shown). We found that depletion of CDC20 led to a transcriptional repression of a central apoptosis inhibitor gene Survivin through up-regulation of p21^WAF1/CIP1^ protein levels. Survivin has been reported highly expressed in many cancers and associated with chemotherapy resistance [61, 62, 63]. As TICs are more resistant to conventional therapy relative to non-TICs [5, 64], CDC20 may also contribute to the radio- or chemo-resistance of TICs in future studies.

Genetic studies of glioblastoma demonstrate common mutational events in growth factor receptor pathways and intracellular mediators that promote cellular proliferation. Our studies suggest that the cellular differentiation state within these malignant cancers may also be reflected in a differential regulation of mitotic control. Our study demonstrates a novel integration of FOXM1, CDC20, and p21^WAF1/CIP1^ with TIC proliferation, survival and tumor growth. With the development of specific CDC20 small molecule inhibitors, such as TAME and Apcin [65, 66], our study may inform the development of novel therapeutic paradigms for glioblastoma and other advanced cancers.

## MATERIALS AND METHODS

### Isolation and culture of cells

Glioblastoma tissues were obtained from excess surgical materials from patients at the Cleveland Clinic after neuropathologist review in accordance with an approved protocol by the Institutional Review Board. To prevent culture-induced drift, patient-derived xenografts were generated and maintained as a recurrent source of tumor cells for study. Immediately upon xenografts removal, a Papain Dissociation System (Worthington Biochemical) was used to dissociate tumors according to the manufacturer's instructions (detailed protocol:http://www.worthington-biochem.com/PDS/default.html). Cells were then cultured in Neurobasal medium supplemented with B27, L-glutamine, sodium pyruvate (Invitrogen), 10 ng/ml basic fibroblast growth factor (bFGF), and 10 ng/ml epidermal growth factor (EGF) (R&D Systems) for at least 6 h to recover surface antigens. No marker is uniformly informative for TICs so we use a combination of functional criteria to validate TICs. Where indicated, TICs and non-TICs were derived immediately after dissociation or after transient xenograft passage in immunocompromised mice using prospective sorting followed by assays to confirm stem cell marker expression, sphere formation, and secondary tumor initiation. Although CD133 is controversial, in the models used in these studies, CD133 has previously identified functional TICs [5]. Therefore, in experiments with matched TIC and non-TIC cultures, we segregated AC133 marker-positive and marker-negative populations using CD133/2-APC conjugated antibody (293C3, Miltenyi Biotech, Auburn, CA) by FACS or magnetic bead separation (Miltenyi), as previously described [5, 11, 13]. The TIC phenotype of these cells was validated by stem cell marker expression (CD133, Olig2, Sox2), functional assays of self-renewal (serial tumorsphere passage), and tumor propagation by *in vivo* limiting dilution.

### Proliferation and neurosphere formation assay

Cell proliferation was measured using Cell-Titer Glow (Promega, Madison, WI). Neurosphere formation was measured by *in vitro* limiting dilution as previously described [11, 13]. All data were normalized to day 0 and presented as mean ± standard deviation.

### Vectors and lentiviral transfection

Lentiviral clones to express shRNA directed against CDC20 (TRCN0000003790, TRCN0000284991), FOXM1 (TRCN0000015544, TRCN0000015546), or a control shRNA insert that does not target human and mouse genes (shCONT, SHC002) were obtained from Sigma-Aldrich (St. Louis, MO). shRNAs with non-overlapping sequences that had the best relative knockdown efficiency were used for all experiments. Tet-on CDC20 expression plasmid was a gift from Dr. Wenyi Wei (Harvard). Lentiviral particles were generated in 293FT cells in stem cell media with co-transfection with the packaging vectors pCMV-dR8.2 dvpr and pCI-VSVG (Addgene) by Lipofectamine 2000 (Invitrogen).

### Western blotting

Cells were collected and lysed in hypotonic buffer with nonionic detergent (50 mM Tris-HCl, pH 7.5; 150 mM NaCl; 0.5% NP-40; 50 mM NaF with protease inhibitors), incubated on ice for 15 minutes and cleared by centrifugation at 10,000 g at 4°C for 10 minutes. Protein concentration was determined using the Bradford assay (Bio-Rad Laboratories, Hercules, CA). Equal amounts of protein were mixed with reducing Laemmli loading buffer, boiled and electrophoresed on NuPAGE Gels (Invitrogen), then transferred to PVDF membranes (Millipore). Blocking was performed for 30 minutes with 5% nonfat dry milk in TBST and blotting performed with primary antibodies for 16 hours at 4°C. Antibodies included CDC20 (Santa Cruz Biotechnology), p21^WAF1/CIP1^ (Cell Signaling), FOXM1 (Santa Cruz Biotechnology) and GAPDH (Sigma).

### *In vitro* limiting dilution assay

For *in vitro* limiting dilution assays, decreasing numbers of cells per well (20, 10, 5, and 1) were plated in 96-well plates. Ten days after plating, the presence and number of neurospheres in each well was quantified. Extreme limiting dilution analysis was performed using software available at http://bioinf.wehi.edu.au/software/elda, as previously described [13].

### Immunofluorescent staining

Cells or 10 μm thick slices of xenografted brain tissue were fixed in 4% paraformaldehyde and immunolabeled using the following antibodies: CDC20 (Santa Cruz Biotechnology, Santa Cruz, CA), OLIG2 (R&D Systems), and SOX2 (R&D Systems). Primary antibodies were incubated overnight at 4°C, followed by species appropriate secondary antibodies (Alexa 488 and 568; Invitrogen Molecular Probes, Eugene, OR) with incubation for 1 hour. Nuclei were stained with DAPI, and slides were then mounted using Fluoromount (Calbiochem, San Diego, CA). Images were taken using a Leica DM4000 Upright microscopy.

### Chromatin immunoprecipitation (ChIP) assay

4 × 10^6^ cells per condition were plated, and ChIP was performed with the CHIP assay kit (Invitrogen, no. 49-2024) following the manufacturer's protocol. Briefly, 5 μg FOXM1 antibody (GeneTex no. GTX102170) or rabbit IgG was used for the immunoprecipitation of the DNA–protein immunocomplexes. Crosslinking was reversed by heating for 6 h at 65°C, followed by digestion with proteinase K. The purified DNA was subjected to quantitative PCR with CDC20-ChIP primers: forward: CCGCTAGACTCTCGTGATAGC, backward: TGGCTCCTTCAAAATCCAAC.

### Intracranial tumor formation

TICs were transduced with lentiviral vectors expressing CDC20 or a non-targeting control (shCONT) shNRA for the knockdown experiments. 36 hours post infection, viable cells were counted and engrafted intracranially into NSG (NOD.Cg-Prkdcscid Il2rgtm1Wjl/SzJ, The Jackson Laboratory, Bar Harbor, ME) mice under a Cleveland Clinic Foundation Institutional Animal Care and Use Committee approved protocol. Animals were then maintained until neurological signs were apparent, at which point they were sacrificed. The brains were harvested and fixed in 4% formaldehyde, cryopreserved in 30% sucrose, and then cryosectioned. Sections were stained with hematoxylin and eosin. In parallel survival experiments, animals were monitored until they developed neurological signs.

## SUPPLEMENTARY FIGURES


