# Supplementary material for: CDC20 maintains tumor initiating cells
Source: Oncotarget. 2015 Apr 28;6(15):13241–54. doi: 10.18632/oncotarget.3676 (PMC4537011; doi:10.18632/oncotarget.3676)
Supplement: Supplementary file 1 [file oncotarget-06-13241-s001.pdf]

SUPPLEMENTARY FIGURES

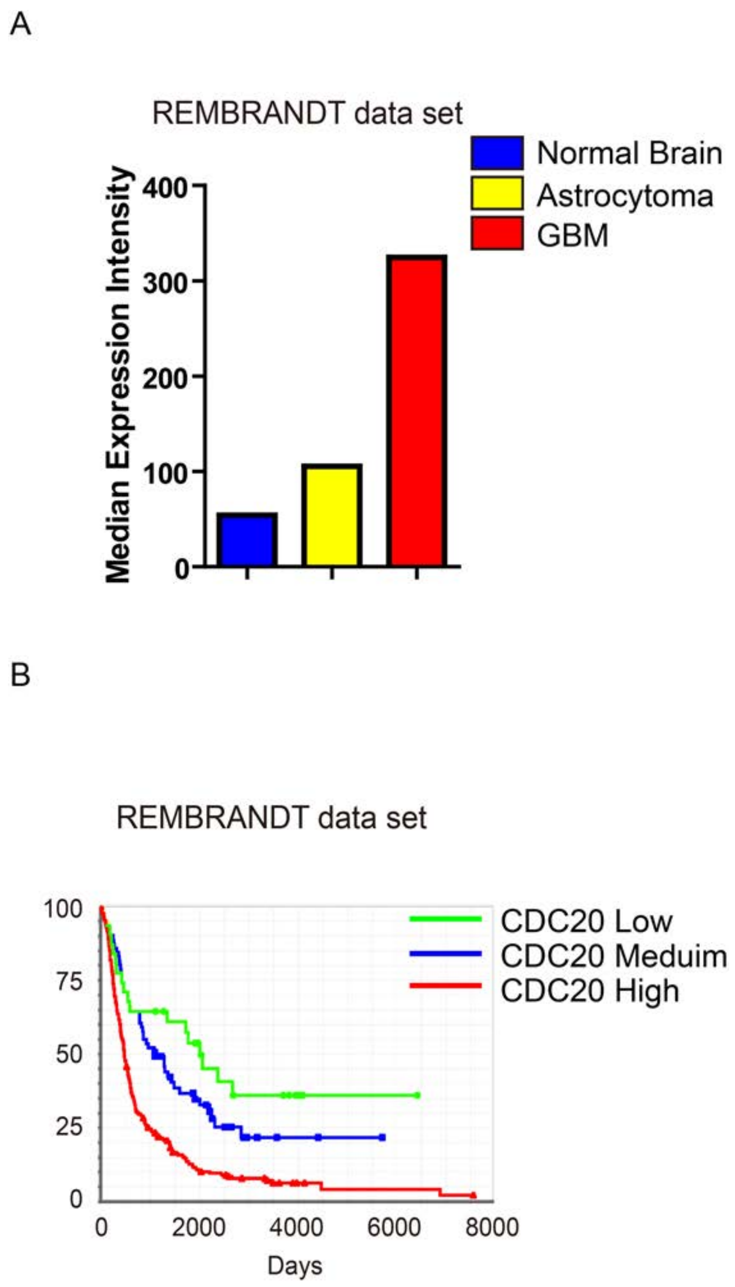

Supplementary Figure S1: CDC20 expression informs patient prognosis. A. Median expression of CDC20 expression in the REMBRANDT dataset in normal or brain tumor types. **B.** Analysis of REMBRANDT data indicates that CDC20 mRNA expression inversely correlates with glioma patient survival (log-rank *p* value: high vs. medium; $p = 6.7 \times 10^{-6}$; high vs. low, $p = 1.3 \times 10^{-5}$)

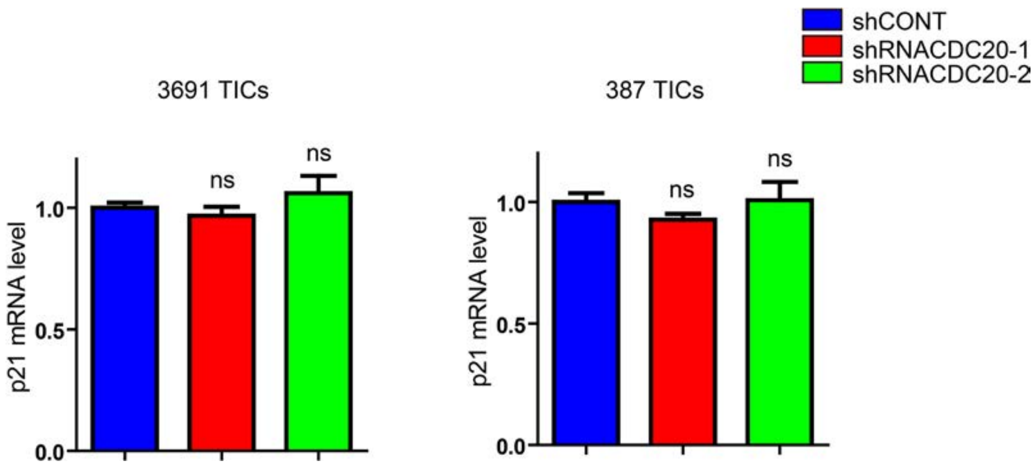

Supplementary Figure S2: CDC20 does not regulate p21$^{WAF1/CIP1}$ mRNA levels. 3691 and 387 TICs were infected with CDC20 shRNAs or shRNA control (shCONT) for 2 days. Total RNA was isolated and cDNA was synthesized by reverse transcription. The mRNA levels of indicated genes were detected by real-time qPCR (*$p < 0.05$; **$p < 0.01$; ***$p < 0.001$; $n = 3$).
